# Phenotypic and genotypic characterization of glycopeptide, aminoglycoside and macrolide resistance among clinical isolates of *Enterococcus faecalis*: a multicenter based study

**DOI:** 10.1186/s13104-019-4339-4

**Published:** 2019-05-27

**Authors:** Mehrdad Zalipour, Bahram Nasr Esfahani, Seyed Asghar Havaei

**Affiliations:** 1 Department of Microbiology, School of Medicine, Isfahan University of Medical Sciences, Hezar Jarib St, Isfahan, Iran; 2 Infectious Diseases and Tropical Medicine Research Center, Isfahan University of Medical Sciences, Isfahan, Iran

**Keywords:** *Enterococcus faecalis*, Antibiotic resistance, *vanA*, *ermB*, *aac (6′)*-*Ie aph (2″)*

## Abstract

**Objectives:**

*Enterococcus faecalis* as part of the normal floras of human gastrointestinal and genitourinary tracts are an important cause of nosocomial infections. The present study aimed to investigate the prevalence of genes encoding antimicrobial resistance and genetic relatedness of clinical isolates of *E. faecalis* among Iranian hospitalized patients.

**Results:**

Antibiotic susceptibility testing results indicated that 53 (22.8%) out of 232 *E. faecalis* isolates were vancomycin resistant (MIC ≥ 256 μg/ml). All of the 53 vancomycin-resistant *E. faecalis* isolates carried the *vanA* and *ermB* genes; whereas *aac (6′)*-*Ie aph (2″)*, *msrA*, and *ermA* gene were found in 96.2%, 30.2% and 3.8% of vancomycin-resistant isolates, respectively. ERIC-PCR typing revealed that 53 vancomycin-resistant isolates were classified into 14 ERIC types. In our results, the high level of resistance to gentamicin, erythromycin and vancomycin in enterococci isolates were mainly related to the presence *of aac (6′)*-*Ie aph (2″), ermB* and *vanA* genes, respectively. Meanwhile, ERIC-PCR analysis demonstrated that most of the evaluated isolates have a close genetic relatedness.

## Introduction

Enterococci are Gram-positive microorganisms and common commensal bacterium of human and animals digestive system [1]. *Enterococcus faecalis*, particularly vancomycin-resistant strains are an important cause of nosocomial infections such as bacteremia, sepsis, endocarditis, urinary tract infection (UTI) and wound infection [1]. The combination of a cell wall active agent (ampicillin, penicillin, or vancomycin) and an aminoglycoside, typically gentamicin has been used frequently for treatment of serious enterococcal infections [2]. However, treatment of enterococcal infections could be difficult due to increasing resistance of enterococci to antimicrobial agents such as b-lactams, high-level resistance to aminoglycosides and more recently to glycopeptides [3].

The emergence and limited therapeutic options of vancomycin-resistant enterococci (VRE) have become a substantial clinical and epidemiological concern since critical ill patients such as patients with end-stage renal disease are at higher risk of colonization and subsequently more complication and treatment cost [4–6]. There are nine types of vancomycin-resistant cluster genes (*van A to vanN)*, that *vanA* and *vanB* possess the greatest clinical significance and are the most commonly reported types in VRE worldwide [7, 8]. High-level resistance to the aminoglycosides usually occurs by the bi-functional aminoglycoside-modifying enzymes (AMEs) with both 6′aminoglycoside acetyltransferase and 2″ aminoglycoside activities, encoded by the structural gene *aac (6′)*-*Ie aph (2″)* which reduce the effect of aminoglycosides, with the exception of streptomycin [9, 10]. The more frequent macrolide resistance determinants in enterococci are ribosomal target modification by 23S rRNA methylases encoded by the erythromycin resistant methylase (*erm*) genes [11, 12]. This modification of the ribosomal target causes crossed resistance to macrolide, lincosamide, streptogramin (MLS) group of antibiotics [12]. The second major macrolide resistance mechanism is active efflux which encoded by the *msrA* or *mefA* genes [13].

Understanding the local molecular epidemiology of VRE is necessary to control the spread of this bacteria in hospital setting. For this purpose, several valuable genotyping methods including ribotyping, pulsed-field electrophoresis (PFGE) and Polymerase Chain Reaction-based techniques are available [14]. Moreover, among DNA-based typing tools, the enterobacterial repetitive intergenic consensus (ERIC)-PCR as a simple, sharp and reproducible typing methods are suitable for local typing of enterococci [15]. Given the importance of vancomycin-resistant *Enterococci* in hospital-acquired infection and there is limited data regarding the molecular properties of VRE isolates in our region, the aim of this study was to determine the frequency of genes encoding antimicrobial resistance and genetic relatedness of clinical isolates of *E. faecalis* among Iranian hospitalized patients.

## Main text

### Methods

In this cross-sectional study during April 2017 to October 2017, a total of 232 *E. faecalis* isolates were collected from specimens of patients hospitalized in four University Teaching Hospitals in Isfahan, central part of Iran. They were obtained from different clinical specimens including urine, wound, blood, tracheal and other body fluids. Identification of the enterococci was performed based on the standard microbiological tests including Gram staining, catalase reaction, growth on Brain Heart Infusion agar with 6.5% NaCl, and bile-esculin test. The *ddlE* gene was targeted using species-specific primers for confirmation of *E. faecalis* isolates as described previously [1].

All of *E. faecalis* isolates were screened for phenotypic susceptibility against 11 antimicrobial agents by disc diffusion method on the Mueller–Hinton Agar (Merck Co., Darmstadt, Germany) based on Clinical and Laboratory Standards Institute (CLSI) guideline [16]. The tested antibiotics (Mast Group Ltd., UK.) were vancomycin (30 μg), teicoplanin (30 μg), erythromycin (15 μg), gentamicin (120 μg), ampicillin (10 μg), ciprofloxacin (5 μg), tetracycline (30 μg), nitrofurantoin (300 μg), rifampin (5 μg), fosfomycin (200 μg) and linezolid (5 μg). Measurement of minimal inhibitory concentration (MICs) of vancomycin was performed by the E-test strips (Liofilchem, Italy) on the Mueller–Hinton Agar accordance with CLSI guideline [16].

PCR was done for detection the *vanA* and *vanB* genes, responsible for resistance to vancomycin, *aac (6′)*-*Ie aph (2″)* gene encoding high level resistance to gentamicin and *ermA, ermB, msrA* and *mefA* genes for macrolide resistance among *E. faecalis* isolates with high level resistance to vancomycin [10, 17]. To do PCR, first bacterial cells were lysed and DNA extraction was done according to the method described by Heidari et al. [15] and was then amplified in 25 μl reaction mixtures containing 2 μl of template DNA, 1 μl of each primer for studied genes, 9 μl of Master Mix, and 12 μl of sterile distilled water. PCR was performed in an Bio-Rad thermocycler with an initial denaturation step of 5 min at 95 °C, followed by 30 cycles of 1 min at 94 °C, 1 min at 50–60 °C according the type of primer and 1 min at 72 °C, and a final extension step of 5 min at 72 °C. The PCR products were analyzed by electrophoresis in 1% agarose gels with 1× TAE (Tris/Acetate/EDTA) buffer and photographed under ultraviolet illumination after staining with safe stain load dye (CinnaGen Co., Iran).

All VRE isolates were selected for analysis by ERIC-PCR and primer sequence used in this study as described previously [18]. To do ERIC-PCR was used of the protocol described in Heidari et al. study [15]. Amplified products were assessed by electrophoresis through 1% agarose gels at 60 V for 3 h in a 0.5× TBE (Tris/Borate/EDTA) buffer and DNA bands were visualized using ultraviolet light after staining with safe stain load dye (CinnaGen Co., Tehran, Iran). ERIC patterns were analyzed using GelJ software, as described previously [20]. Isolates with a similarity coefficient equal or above 90% were clustered as the same genotypes.

Differences in the frequency of resistance genes and antimicrobial resistance pattern between selected *E. faecalis* isolates were analysed using the Chi-square test for each antimicrobial agent. A difference was considered statistically significant if the P-value was less than 0.05.

### Results

During 6 months of the study, a total of 232 *E. faecalis* isolates were isolated from different clinical specimens. Overall, 70% of isolates were collected from urine samples and 30% from other clinical samples. About 45% of *E. faecalis* isolates were isolated from male and 55% from female patients.

Among 232 *E. faecalis* isolates, the highest antibiotic resistance was seen against tetracycline (93.5%) followed by erythromycin (87%), and ciprofloxacin (80%). None of the isolates was found to be resistant to fosfomycin and linezolid. Antibiotic susceptibility testing results indicated that 53 (22.8%) isolates were vancomycin resistant (MIC ≥ 256 μg/ml). More than half of the isolates (52.5%) were high-level gentamicin resistance. In addition, the antibiotic resistance against vancomycin, teicoplanin, ampicillin and gentamicin were significantly higher among vancomycin-resistant *E. faecalis* than vancomycin-susceptible *E. faecalis* (P < 0.001). Moreover, the full results of antibiotic resistance pattern and comparison of the susceptibility patterns of vancomycin-resistant *E. faecalis* and vancomycin-susceptible *E. faecalis* to antibiotics are presented in Table 1.

**Table 1:** Antibiotic resistance pattern of *Enterococcus faecalis* isolates, no. (%)

Antimicrobial agent	No. (%) of vancomycin-resistant isolates (N = 53) (%)			No. (%) of vancomycin-susceptible isolates (N = 179) (%)			Total no. (%) of isolates (N = 232) (%)		
	S	I	R	S	I	R	S	I	R
Teicoplanin	2 (3.8)	0 (0)	51 (96.2)	174 (97.2)	5 (2.8)	0 (0)	176 (75.8)	5 (2.2)	51 (22.0)
Ampicillin	22 (41.5)	–	31 (58.5)	146 (81.5)	–	33 (18.5)	168 (72.4)	–	64 (27.6)
Tetracycline	5 (9.5)	0 (0)	48 (90.5)	10 (5.5)	0 (0)	169 (94.5)	15 (6.5)	0 (0)	217 (93.5)
Ciprofloxacin	1 (1.8)	2 (3.8)	50 (94.4)	9 (5.0)	35 (19.5)	135 (75.5)	10 (4.0)	37 (16.0)	185 (80.0)
Erythromycin	0 (0)	0 (0)	53 (100)	5 (2.7)	25 (14.0)	149 (83.3)	5 (2.3)	25 (10.7)	202 (87.0)
Nitrofurantoin	43 (81.2)	0 (0)	10 (18.8)	155 (86.5)	0 (0)	24 (13.5)	198 (85.3)	0 (0)	34 (14.7)
Rifampin	16 (30.2)	13 (24.5)	24 (45.3)	69 (38.5)	39 (21.8)	71 (39.7)	85 (36.6)	52 (22.4)	95 (41.0)
Gentamicin	10 (18.9)	0 (0)	43 (81.1)	102 (57.0)	0 (0)	77 (43.0)	110 (47.5)	0 (0)	122 (52.5)
Linezolid	53 (100)	0 (0)	0 (0)	179 (100)	0 (0)	0 (0)	232 (100)	0 (0)	0 (0)
Fosfomycin	53 (100)	0 (0)	0 (0)	179 (100)	0 (0)	0 (0)	232 (100)	0 (0)	0 (0)

All of the 53 vancomycin-resistant *E. faecalis* isolates carried the *vanA* gene, whereas the *vanB* gene was not seen in any of this isolates. Also, *aac (6′)*-*Ie aph (2″)* gene was found in 96.2% of vancomycin-resistant isolates. The results of the amplification of erythromycin encoding genes showed that all vancomycin-resistant isolates were positive for *ermB*, whereas *ermA* and *msrA* genes were found 3.8% and 30.2%, respectively. Meanwhile, *mef* A gene was not found in any of the vancomycin-resistant isolates. The coexistence of *ermB* and *ermA* among vancomycin-resistant isolates were 3.8%. The resistance genes distribution and resistance patterns among vancomycin-resistant *E. faecalis* are shown in Table 2.

**Table 2:** The distribution of resistance genes and resistance patterns among vancomycin-resistant *Enterococcus faecalis* isolates

No. of isolates	Male (M) Female (F)	Source	Ward	Resistance patterns	Resistance genes	ERIC types
1	M	Urine	Infectious diseases	AMP, TEC, VAN, CIP, E, TET, GEN	*VanA, ermB, aac (6′)*-*Ie aph (2″)*	*G*
3	M	Urine	ICU	TEC, VAN, CIP, E, TET, GEN	*VanA, ermB, aac (6′)*-*Ie aph (2″)*	*A*
5	M	Catheter	ICU	TEC, VAN, CIP, E, TET, GEN	*VanA, ermB, aac (6′)*-*Ie aph (2″)*	*A*
6	F	Urine	Emergency	TEC, VAN, CIP, E, TET, GEN	*VanA, ermB, aac (6′)*-*Ie aph (2″)*	E
8	F	Urine	Outpatient	AMP, TEC, VAN, E, TET, GEN	*VanA, ermB, aac (6′)*-*Ie aph (2″)*	*A*
10	F	Wound	Surgery	RIF, TEC, VAN, CIP, E, TET, GEN	*VanA, ermB, aac (6′)*-*Ie aph (2″)*	*A*
15	M	Blood	Infectious diseases	AMP, RIF, TEC, VAN, CIP, E, TET, GEN	*VanA, ermB, msrA, aac (6′)*-*Ie aph (2″)*	*A*
16	M	Urine	Emergency	AMP, TEC, VAN, CIP, E, TET, GEN	*VanA, ermB, msrA, aac (6′)*-*Ie aph (2″)*	*A*
18	M	Urine	Internal	TEC, VAN, CIP, E, TET, GEN	*VanA, ermB, aac (6′)*-*Ie aph (2″)*	*A*
25	F	Urine	Emergency	TEC, VAN, E, TET	*VanA, ermA, ermB*	*Single*
29	F	Blood	Respiratory	AMP, RIF, TEC, VAN, CIP, E, TET, GEN	*VanA, ermB, aac (6′)*-*Ie aph (2″)*	*C*
30	F	Urine	Internal	AMP, RIF, TEC, VAN, CIP, E, TET, GEN, FM	*VanA, ermB, aac (6′)*-*Ie aph (2″)*	*D*
31	F	Urine	Internal	AMP, TEC, VAN, CIP, E, TET, GEN, FM	*VanA, ermB, aac (6′)*-*Ie aph (2″)*	*C*
32	M	Urine	Internal	AMP, RIF, VAN, CIP, E, TET, GEN	*VanA, ermB, msrA, aac (6′)*-*Ie aph (2″)*	*A*
35	F	Urine	ICU	AMP, TEC, VAN, CIP, E, TET, FM	*VanA, ermB, msrA, aac (6′)*-*Ie aph (2″)*	*A*
40	F	Urine	Emergency	TEC, VAN, CIP, E, TET, GEN	*VanA, ermB, aac (6′)*-*Ie aph (2″)*	*Single*
41	F	Urine	Surgery	AMP, RIF, TEC, VAN, CIP, E, TET, GEN	*VanA, ermB, aac (6′)*-*Ie aph (2″)*	*A*
44	F	Urine	Rheumatology	TEC, VAN, CIP, E, TET	*VanA, ermB, aac (6′)*-*Ie aph (2″)*	*A*
51	M	Urine	ICU	AMP, TEC, VAN, CIP, E, TET, GEN	*VanA, ermB, msrA, aac (6′)*-*Ie aph (2″*	*A*
52	M	Urine	Surgery	TEC, VAN, CIP, E, TET GEN	*VanA, ermB, aac (6′)*-*Ie aph (2″*	*A*
54	F	Tracheal	ICU	AMP, TEC, VAN, CIP, E, TET, GEN	*VanA, ermB, msrA, aac (6′)*-*Ie aph (2″)*	*A*
66	F	Wound	Rheumatology	TEC, VAN, E, TET	*VanA, ermB, aac (6′)*-*Ie aph (2″)*	*Single*
67	F	Urine	Urology	AMP, RIF, TEC, VAN, CIP, E, TET, GEN	*VanA, ermB, aac (6′)*-*Ie aph (2″)*	*Single*
70	M	Wound	Internal	AMP, TEC, VAN CIP, E TET	*VanA, ermB, aac (6′)*-*Ie aph (2″)*	*D*
72	M	Urine	ICU	AMP, RIF, VAN, CIP, E, TET, GEN	*VanA, ermB, aac (6′)*-*Ie aph (2″)*	*F*
78	F	Urine	NICU	AMP, RIF, TEC, VAN, CIP, E, TET, GEN	*VanA, ermB, aac (6′)*-*Ie aph (2″)*	*G*
79	M	Wound	Infectious diseases	AMP, RIF, TEC, VAN, CIP, E, TET, GEN	*VanA, ermA, ermB, aac (6')-Ie aph (2'')*	*A*
87	F	Eye	NICU	TEC, VAN, CIP, E, TET	*VanA, ermB, aac (6′)*-*Ie aph (2″)*	*F*
90	M	Tracheal	ICU	TEC, VAN, CIP, E, TET, GEN	*VanA, ermB, aac (6′)*-*Ie aph (2″)*	*A*
92	F	Urine	Internal	AMP, RIF, TEC, VAN, CIP, E	*VanA, ermB, aac (6′)*-*Ie aph (2″)*	*B*
101	F	Tracheal	ICU	RIF, TEC, VAN, CIP, E, TET, GEN	*VanA, ermB, aac (6′)*-*Ie aph (2″)*	*Single*
102	F	Urine	*Respiratory*	AMP, RIF, TEC, VAN, CIP, E, GEN, FM	*VanA, ermB, aac (6′)*-*Ie aph (2″)*	*B*
103	F	Urine	ICU	TEC, VAN, CIP, E, TET, GEN	*VanA, ermB, aac (6′)*-*Ie aph (2″)*	*A*
113	M	Chest	Surgery	AMP, RIF, TEC, VAN, CIP, E, GEN	*VanA, ermB, msrA, aac (6′)*-*Ie aph (2″)*	*B*
115	M	Urine	ICU	AMP, RIF, TEC, VAN, CIP, E, TET, GEN, FM	*VanA, ermB, aac (6′)*-*Ie aph (2″)*	*B*
117	M	Urine	Internal	AMP, RIF, TEC, VAN, CIP, E, TET, GEN, FM	*VanA, ermB, msrA, aac (6′)*-*Ie aph (2″)*	*B*
118	M	Abscess	Internal	AMP, TEC, VAN, CIP, E, TET, GEN	*VanA, ermB, msrA, aac (6′)*-*Ie aph (2″)*	*A*
122	M	Abscess	ICU	AMP, RIF, TEC, VAN, CIP, E, TET, GEN, FM	*VanA, ermB, aac (6′)*-*Ie aph (2″)*	*A*
144	F	Tracheal	Internal	RIF, TEC, VAN, CIP, E, TET, GEN	*VanA, ermB, msrA, aac (6′)*-*Ie aph (2″)*	*A*
145	F	Urine	ICU	AMP, TEC, VAN, CIP, E	*VanA, ermB, msrA,*	*B*
146	M	Urine	Emergency	AMP, RIF, TEC, VAN, CIP, E, TET, GEN, FM	*VanA, ermB, msrA, aac (6′)*-*Ie aph (2″)*	*D*
156	F	Urine	Internal	AMP, TEC, VAN, CIP, E, TET, GEN, FM	*VanA, ermB, aac (6′)*-*Ie aph (2″)*	*A*
160	F	Urine	ICU	AMP, RIF, TEC, VAN, CIP, E, TET, GEN	*VanA, ermB, msrA, aac (6′)*-*Ie aph (2″)*	*Single*
168	F	CSF	CCU	TEC, VAN, CIP, E, TET, GEN	*VanA, ermB, aac (6′)*-*Ie aph (2″)*	*C*
169	F	Urine	CCU	TEC, VAN, CIP, E, TET, GEN	*VanA, ermB, msrA, aac (6′)*-*Ie aph (2″)*	*C*
184	F	Abscess	Internal	AMP, RIF, TEC, VAN, CIP, E, TET, GEN	*VanA, ermB, aac (6′)*-*Ie aph (2″)*	*Single*
185	M	Urine	ICU	TEC, VAN, CIP, E, TET, GEN	*VanA, ermB, msrA, aac (6′)*-*Ie aph (2″)*	*A*
186	M	Urine	Internal	AMP, TEC, VAN, CIP, E, TET, GEN	*VanA, ermB, msrA, aac (6′)*-*Ie aph (2″)*	E
187	F	Blood	ICU	TEC, VAN, CIP, E, TET	*VanA, ermB, aac (6′)*-*Ie aph (2″)*	*A*
191	F	Urine	Internal	TEC, VAN, CIP, E, TET, GEN	*VanA, ermB, aac (6′)*-*Ie aph (2″)*	*A*
196	M	Urine	Surgery	RIF, TEC, VAN, CIP, E, TET	*VanA, ermB, aac (6′)*-*Ie aph (2″)*	*A*
198	F	Urine	Internal	RIF, TEC, VAN, CIP, E, TET, GEN	*VanA, ermB, aac (6′)*-*Ie aph (2″)*	*A*
220	M	Urine	Emergency	AMP, RIF, TEC, VAN, CIP, E, TET, GEN, FM	*VanA, ermB, aac (6′)*-*Ie aph (2″)*	*A*

**Fig. 1:**
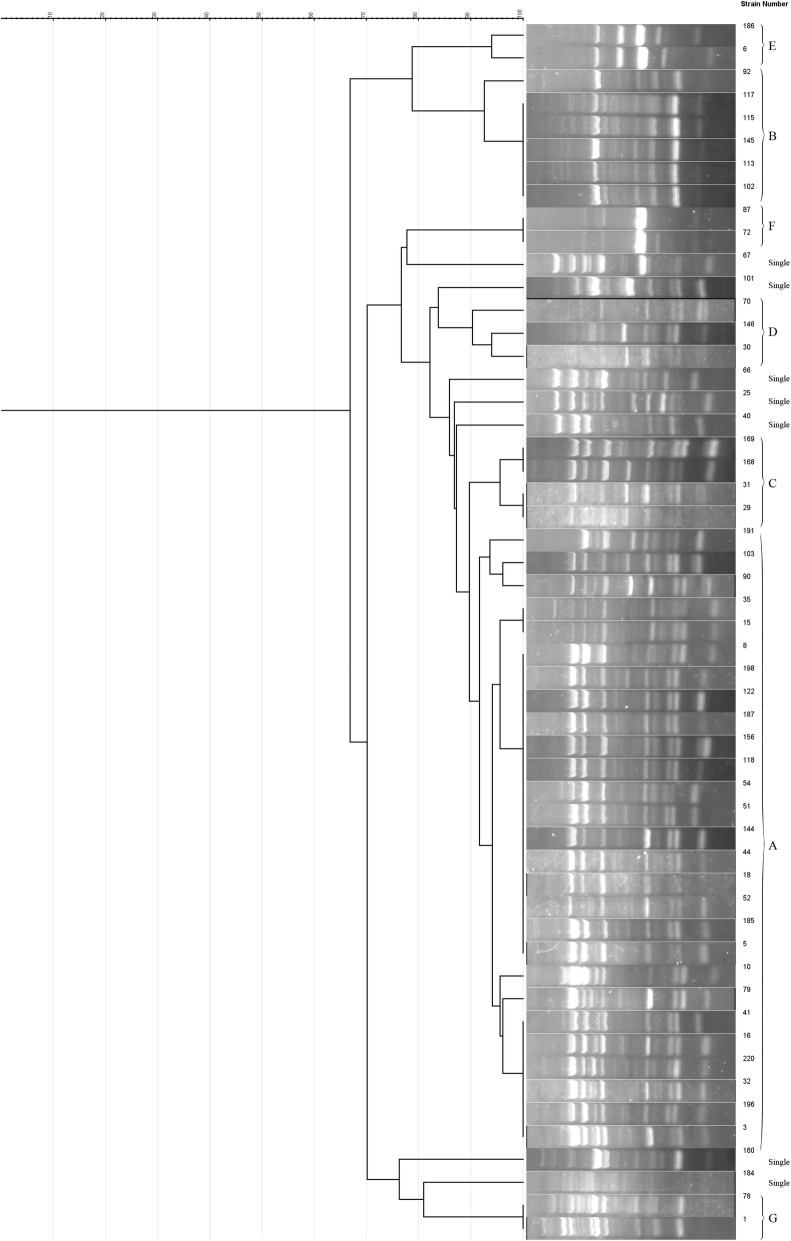
Dendrogram showing relatedness between ERIC-PCR patterns of 53 vancomycin-resistant *Enterococcus faecalis* strains

Dendogram and Gel electrophoresis image of ERIC-PCR products from *E. faecalis* strains was showed in Fig. 1. The number of bands was varied from 3 to 10 bands and the size ERIC fragments ranged from 100 bp to 1.5 kb. ERIC-PCR typing revealed that 53 vancomycin-resistant isolates were classified into 14 ERIC types according to 90% cut off. The predominant type was A which containing 27 isolates. Moreover, six isolates were clustered in genotype B, followed by C type (four), D (three), E (two), F (two), G (two isolates) and other isolates were distributed in scattered patterns and showed 7 single types (Fig. 1). According to our results, 46 (86.8%) isolates were classified into 7 main genotypes (A–G). However, our study results showed that most of the examined strains have a close genetic relatedness. The heterogeneity amongst the isolates obtained from UTIs was more than other infections (Table 2).

### Discussion

In recent decade’s enterococci, especially *E. faecalis* have been emerged as an important cause of healthcare-associated infections [1]. In the present study, more than 22% of isolated *E. faecalis* were vancomycin-resistant but the results of recent meta-analysis studies in Iran have revealed lower rates of VRE isolation from clinical specimens [20]. Moreover, increasing VRE prevalence among hospitalized patients was also reported previously from several studies in the country [8, 21]. In this study, all VRE isolates carried *vanA* gene and none of them has *vanB* gene. Similarly, previous researches had indicated that the *vanA* gene is typically responsible for high-level resistance to glycopeptides in the *E. faecalis* isolates [7, 21–23]. In contrast to our results, Samadi et al. in Tabriz and Rengaraj et al. of India showed that the *vanB* genotype is the predominant type of vancomycin resistance in *E. faecalis* isolates [8, 24]. Resistance to vancomycin in enterococci could lead to the appearance of multidrug-resistant strains resulting in failure of antimicrobial therapy with increased morbidity and mortality in patients [4]. Moreover, Transfer of the *vanA* gene cluster from Enterococcus species to other gram-positive pathogens such as *Staphylococcus aureus* is a very important phenomenon as increasing the public health concern [25]. Consistent with previous studies conducted in the Saudi Arabia and Ethiopia, in this study majority of the *E. faecalis* isolates (80%) and all of vancomycin-resistant isolates had an MDR pattern and more than 55% VRE isolates were resistant to ≥ 6 tested antibiotics [26, 27].

In our results, 52.5% of *E. faecalis* isolates were High-level gentamicin resistance (HLGR) and gentamicin resistance is more prevalent among clinical vancomycin-resistant *E. faecalis* isolates compared to vancomycin-susceptible *E. faecalis*. This finding is in accordance with previous studies in Iran and Kuwait [17, 28, 29] and was, in contrast, to report from Turkey [30]. The results of this study showed that 96.2% of vancomycin-resistant isolates and all HLGR isolates carried *aac (6′)*-*Ie aph (2″)* resistance gene. These results were in consistent with previous Iranian studies that showed high rate of HLGR enterococci contained the *aac (6′)*-*Ie aph (2″)* gene [7, 10, 15].

In the present study, more than 87% all of *E. faecalis* strains and 100% of vancomycin-resistant isolates were resistance to erythromycin. Decrease the effect of erythromycin on enterococci probably is due to the widespread use of macrolides family. In this study, similar to numerous studies indicated that *ermB* gene plays a predominant role in the development of MLS_B_ phenotype among enterococci, the *ermB* gene was present in the all vancomycin-resistant isolates which were resistance to erythromycin [15, 17, 31]. But contrary to previous studies, in the present study, the investigated gene encoding efflux pump *msrA* was found in the 30.2% of VRE strains and *mefA* was not found in any of the isolates [13, 15].

Analysis of banding profiles of ERIC-PCR result showed most of the evaluated isolates have a close genetic relatedness. In this study, a total of 14 different ERIC profiles were observed among 53 VRE isolates. The strains that classified in the same ERIC types relatively presented similar drug resistance pattern. In our study, the majority of the isolates (27/53) were clustered in A type and most of isolates in these type were isolated from the same hospital ICU or internal ward and collected from urine and showed similar antibiotics resistance patterns. These data may suggest that an influence of epidemiological relatedness on the clustering of *E. faecalis* circulating strains in Isfahan city, as four clusters of VRE strains with high relatedness were recovered from the same period of isolation and location. This indicates the horizontal transfer of resistance genes among different types of *E. faecalis* isolates in hospital. Heterogeneity among isolates may contribute to facilitating survival of various enterococci strain in the environment of hospital. However, resistance to antimicrobial agents in such strains may lead to colonization and also enhancing potential spread from person to person in hospital setting.

In conclusion, the high incidence of antibiotic resistance in VRE isolates in our study can be viewed as one of the major public health crisis because the control of infections resulting from these resistant bacteria are difficult. This study demonstrated that high-level resistance to gentamicin, erythromycin and vancomycin in enterococci isolates were mainly related to the presence *of aac (6′)*-*Ie aph (2″), ermB* and *vanA* genes, respectively. The ERIC-PCR analysis demonstrated that the evaluated isolates were relatively heterogeneous and this may causes problems for the treatment of infections due to *E. faecalis* strains in hospitals.

## Limitation

One limitation of this study is the apparently small number of enterococci isolates, especially VRE strains that were investigated for virulence and antibiotic resistance determinants. Second, to identification of the source for pathogen transmission and take preventive measures in hospital setting, molecular analysis of environmental specimens was required.

## Data Availability

The datasets used and/or analyzed during the current study are available from the corresponding author on reasonable request.

## Abbreviations

AMEs: aminoglycoside-modifying enzymes
ERIC: enterobacterial repetitive intergenic consensus
*erm*: erythromycin resistant methylase
UTI: urinary tract infection
PCR: polymerase chain reaction
PFGE: pulsed-field electrophoresis
VRE: vancomycin-resistant enterococci
HLGR: high-level gentamicin resistance
